# Vectors for Glioblastoma Gene Therapy: Viral & Non-Viral Delivery Strategies

**DOI:** 10.3390/nano9010105

**Published:** 2019-01-16

**Authors:** Breanne Caffery, Jeoung Soo Lee, Angela A. Alexander-Bryant

**Affiliations:** 1 Drug Design, Development, and Delivery (4D) Laboratory, Clemson University, Clemson, SC 29634, USA; bhourig@g.clemson.edu (B.C.); ljspia@clemson.edu (J.S.L.); 2 Nanobiotechnology Laboratory, Department of Bioengineering, Clemson University, Clemson, SC 29634, USA

**Keywords:** glioblastoma multiforme, gene therapy, viral vector, non-viral vector, gene delivery, siRNA

## Abstract

Glioblastoma multiforme is the most common and aggressive primary brain tumor. Even with aggressive treatment including surgical resection, radiation, and chemotherapy, patient outcomes remain poor, with five-year survival rates at only 10%. Barriers to treatment include inefficient drug delivery across the blood brain barrier and development of drug resistance. Because gliomas occur due to sequential acquisition of genetic alterations, gene therapy represents a promising alternative to overcome limitations of conventional therapy. Gene or nucleic acid carriers must be used to deliver these therapies successfully into tumor tissue and have been extensively studied. Viral vectors have been evaluated in clinical trials for glioblastoma gene therapy but have not achieved FDA approval due to issues with viral delivery, inefficient tumor penetration, and limited efficacy. Non-viral vectors have been explored for delivery of glioma gene therapy and have shown promise as gene vectors for glioma treatment in preclinical studies and a few non-polymeric vectors have entered clinical trials. In this review, delivery systems including viral, non-polymeric, and polymeric vectors that have been used in glioblastoma multiforme (GBM) gene therapy are discussed. Additionally, advances in glioblastoma gene therapy using viral and non-polymeric vectors in clinical trials and emerging polymeric vectors for glioma gene therapy are discussed.

## 1. Introduction

Glioblastoma multiforme (GBM) is a type of glioma that arises from astrocytes, defined by the World Health Organization (WHO) as a grade IV glioma [1,2]. GBM is not only the most common malignant primary brain tumor, but also the most aggressive of malignant tumors, with recurrence in nearly all patients [3]. GBM affects about three people out of every 100,000 per year, accounting for approximately 15% of primary brain tumors, and 80% of malignant primary brain tumors. GBM is about two times more common in whites than in blacks, and 1.5 times more common in men than in women, with an average age at onset of 64 [4].

High-grade gliomas are typically located in undesirable locations in the cerebral hemisphere and are classified as diffuse gliomas due to their high rate of infiltration into surrounding brain tissue. These factors allow for persistent tumor growth and lessen the chance of remission, with progression to grade III or IV gliomas likely even in most low-grade diffuse gliomas [2,5]. The current standard of care for treating GBM includes surgical resection of the tumor, radiation therapy, and chemotherapy via temozolomide (TMZ) [6]. Carmustine (BCNU, Gliadel™) wafers have been used as local adjuvant therapy in combination with systemic TMZ since its approval; however, its use has been limited due to observed toxicities and ambiguity of overall survival benefit [7,8]. Additionally, bevacizumab, a monoclonal antibody that inhibits vascular endothelial growth factor (VEGF), is used for the treatment of recurrent glioblastomas [9]. However, even with aggressive treatment, survival rates remain between 12 and 15 months, and the 3-year survival rate is less than 16% [1,10]. GBM remains an essentially incurable disease, resulting in a patient death rate of greater than 95% within five years of diagnosis [11]. Consequently, there is a clear need for advancement in treatment strategies to improve outcomes for patients with GBM. Gene therapy may provide a viable alternative to conventional treatments towards combating cancer progression in GBM. 

This review discusses gene expression in GBM, the limitations to conventional therapy, and current approaches to circumvent these barriers using gene therapy. Advances in gene delivery systems will be reviewed, highlighting viral and non-viral vectors used for GBM gene therapy. Trials bringing these therapies closer to clinical approval to date will be discussed, as well as preclinical studies, particularly using polymeric nanoparticles, which have shown promise as future vectors for delivery of gene therapy in GBM patients.

## 2. Gene Expression in GBM

Glioma occurs due to sequential acquisition of genetic alterations, causing a transformation from benign to malignant tissue [12]. Glioblastoma can occur in four clinical subtypes including classical, proneural, mesenchymal and neural GBM [13]. Classical, or primary GBM arises *de novo,* and occurs in about 95% of cases, only requiring about 3–6 months to develop [1]. Proneural or secondary GBM arises as a recurrence from a previous anaplastic or low-grade astrocytoma, usually requiring 10–15 years to develop [14]. Classical GBM can be identified by chromosome 7 amplification paired with chromosome 10 loss, as well as by increased expression of the epidermal growth factor receptor (EGFR) and mutations in phosphatase and tensin homologue (PTEN) [1,13]. In a study conducted by Verhaak et al., point or vIII EGFR mutations were found in over half of GBM cases analyzed [13]. EGFR overexpression, observed in 97% of patients with classical GBM, causes a reduction in apoptosis and increased proliferation through the Ras-Shc-Grb2 pathway, causing uncontrolled cell growth [14]. PTEN is a tumor suppressor, and when mutated, the loss of function causes activation of the P13K/Akt/mTOR pathway, leading to proliferation, growth, and migration [15]. Disrupted regulation of this pathway has been shown to contribute to tumorigenesis and resistance in various cancers [16]. Deletion of CDKN2A, coding for tumor suppressor p16INK4A, was also significantly associated with the classical subtype. 

Proneural GBM often presents with increased expression of platelet-derived growth factor receptor alpha (PDGFRA), as well as mutated tumor suppressor p53, isocitrate dehydrogenase 1 (IDH-1), and retinoblastoma genes [11,13,14]. PDGFRA is mitosis-promoting, and overexpression of this mitogen promotes tumor cell proliferation [14]. IDH-1 point mutations were found to occur in about 30% of proneural cases [14]. Mutation of IDH alters DNA and histone methylation and is often found in the early development of diffuse gliomas [17,18]. The p53 gene normally functions as a switch to turn on G1 cell cycle arrest or apoptosis, regulating cell growth [14]. Overexpression of p53 has been shown to negatively regulate MGMT transcription, suggesting that repair of wild-type p53 may increase therapeutic efficacy in GBM therapy [19]. Interestingly, p53 mutations have been found in 54% of proneural GBM but are almost never observed in classical GBM [13]. More recently, interferon-β (IFN-β) has been found to sensitize T98G GBM cells to TMZ, which was also thought to be a function of induced p53 overexpression [20]. 

Mesenchymal GBM presents most prominently with deletion or mutations of the tumor suppressor gene, neurofibromin 1 (NF-1). Similar to the proneural subtype, p53 and mutations occur in about 32% of mesenchymal cases [13]. Genes in the tumor necrosis factor superfamily are also overexpressed, correlating with the high degree of necrosis observed [13]. Mesenchymal GBM also shows characteristics of epithelial-to-mesenchymal transition (EMT) with high expression of mesenchymal and astrocytic markers, such as CD44 and MERTK [13]. EMT in GBM may be induced by hypoxia [21] or upstream regulators of EMT, including TGF-β [21] and S100A4 [22].

A fourth subtype of GBM, the neural subgroup, has been classified due to its similarity in gene expression to normal neurons or nerve cells. Neural GBM presents with mutations similar to the other subgroups with no outstanding genetic amplification or mutation rates that would differentiate the neural subgroup from the other subgroups [13]. In advanced strategies for treating GBM patients, evaluating the expression of key genes may allow for selection of more personalized and effective therapies. Gene targets in GBM are summarized in Table 1.

## 3. Barriers to Drug and Gene Delivery

**Figure 1:**
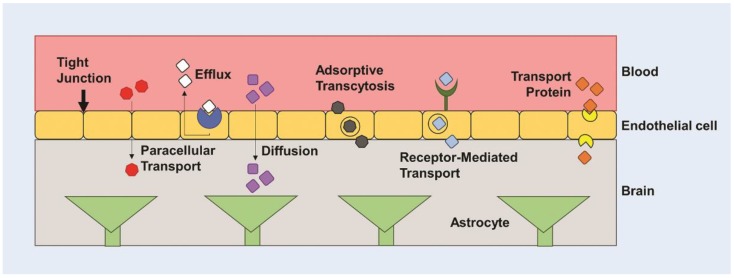
The blood brain barrier (BBB). The BBB regulates entry of nutrients to the brain and prevents entry of cells and large molecules via tight junctions. There are several mechanisms for transporting molecules across the BBB, including paracellular transport, diffusion, protein transporters, receptor-mediated transport, and adsorptive transcytosis.

Various drugs including TMZ, BCNU, and cisplatin have been used in patients with GBM, but several barriers limit effective treatment, including inefficient delivery across the blood-brain barrier (BBB) and chemotherapeutic resistance. The BBB is a cellular barrier that regulates ionic concentrations to allow for synaptic signaling in the brain, while also preventing entry of cells and large molecules via tight junctions between endothelial cells (Figure 1) [5,23]. It exists to regulate the transport of essential nutrients to the brain and to protect the brain from neurotoxins. It is estimated that less than 2% of small molecule drugs and no large molecule drugs or genes are able to cross the BBB [24,25]. It has been found that drugs greater than 400 Da are not often able to cross the BBB. However, this is not a finite cutoff, as peptides greater than 600 Da are known to cross the BBB with relative ease [26], and a 7800 Da molecule, cytokine-induced neutrophil chemoattractant-1 (CNC1), is able to cross the BBB through transmembrane diffusion [27]. Crossing the BBB is highly dependent on many other factors, such as charge, molecular weight, and hydrophobicity, creating a non-linear relationship between size and ability to traverse the BBB [28,29]. Molecules can cross the BBB through various active transport mechanisms, including carrier mediated influx or efflux, and through passive transport mechanisms, including transmembrane diffusion, and paracellular transport (Figure 1) [28,30]. Most molecules that are able to traverse the BBB do so through transmembrane diffusion or active transport. Low molecular weight lipid-soluble molecules are favorable for passive diffusion, whereas water-soluble molecules tend to traverse using active transport processes, including adsorptive- or receptor-mediated transport [26]. The BBB has been a consistent challenge in creating effective delivery systems for therapeutics. Many nanoparticle (NP) delivery systems are designed to rely on diffusion and passive targeting of tumor tissue via the enhanced permeability and retention (EPR) effect [30,31]. Fenestrated capillaries or a dysfunctional endothelium exist in areas of rapidly grown and poorly developed vessels due to increased vascular endothelial growth factor (VEGF) expression and angiogenesis [32,33]. The leaky nature of the vasculature creates an interrupted blood-brain tumor barrier, which may allow for increased therapeutic concentrations in the glioma tissue. However, the EPR effect may be inefficient in therapeutic delivery to brain tumors due to the density of the tumor matrix and the increased interstitial fluid pressure inhibiting both diffusion and convective transport [34]. Furthermore, glioma cells tend to easily travel outside the tumor to other normal regions of the brain. This metastasis not only makes the glioma more difficult to treat, but also reduces the quantity of therapeutic reaching tumor cells in the intact regions of the brain with perfectly functioning BBB [5].

**Figure 2:**
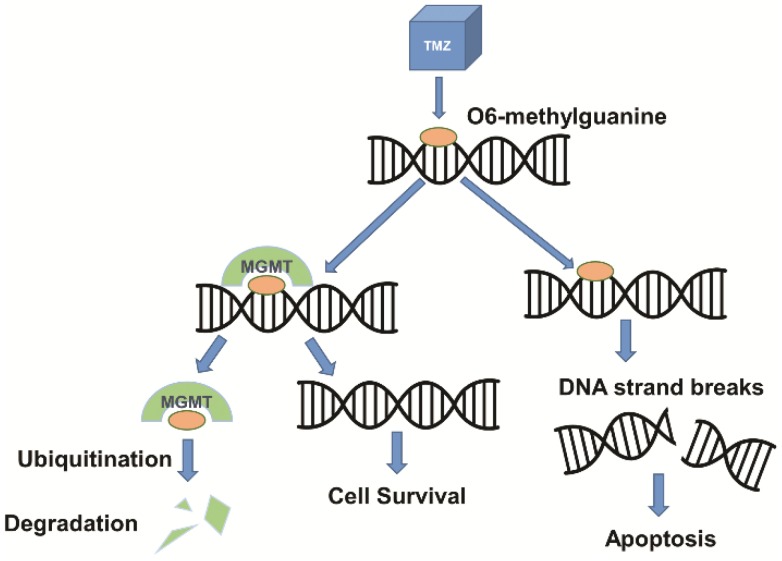
Mechanisms of TMZ and MGMT in DNA damage and repair. TMZ, a DNA alkylating agent, methylates DNA at the O6 position of guanine, resulting in DNA damage and apoptosis of tumor cells. MGMT, a DNA repair protein, removes alkyl adducts from the O6 position of guanine, inhibiting the potentially therapeutic effect of TMZ.

Drug resistance is another major barrier in the treatment of GBM due to overexpression of drug resistance genes. Additional protection of the BBB exists in the form of various efflux transport systems which remove unwanted substances that are able to traverse the BBB. A largely studied efflux pump, P-glycoprotein or multidrug resistance protein 1 (MDR1), encoded by the ATP-binding cassette sub-family B member 1 (ABCB1) gene, has been a persistent challenge in therapeutic delivery due to its efficacy in removing small molecules from the brain [26]. A wide variety of ATP-dependent substrates are recognized by ABCB1, allowing for resistance to occur when therapeutics are recognized and pumped out of the cell through the efflux pump, reducing cytotoxicity and drug efficacy [35,36]. An MDR1a knockout study demonstrated that P-glycoprotein is a major impediment for drug passage through the BBB, after finding significantly increased drug concentrations in the brains of P-glycoprotein knockout mice [37]. Drug resistance in GBM patients has also been attributed in part to overexpression of the (O)6-methylguanine-DNA- methyltransferase (MGMT) gene. The MGMT gene codes for a protein that removes alkyl adducts at the O(6) position of guanine as a natural repair mechanism to prevent apoptosis due to DNA methylation [38]. Although it is a natural process for DNA repair, when MGMT is upregulated in tumor cells, this mechanism allows for drug resistance when treating GBM with temozolomide (TMZ). TMZ alkylates DNA at the O(6) position of guanine in order to cause DNA damage and programmed cell death [39]. In this case, the damage done by TMZ is possibly reversed due to epigenetic or drug-induced upregulation of MGMT in GBM cells (Figure 2). MGMT methylation status was the first predictive biomarker identified in glioma patients [40]; additionally, Hegi et al. found that epigenetic silencing of MGMT was correlated with longer patient survival when treated with alkylating agents and radiotherapy [39]. In attempts to overcome current barriers to effective treatment, delivery systems for gene and drug therapies have been researched and tested in vivo and/or in the clinic.

## 4. Vectors for Glioblastoma Gene Therapy

**Figure 3:**
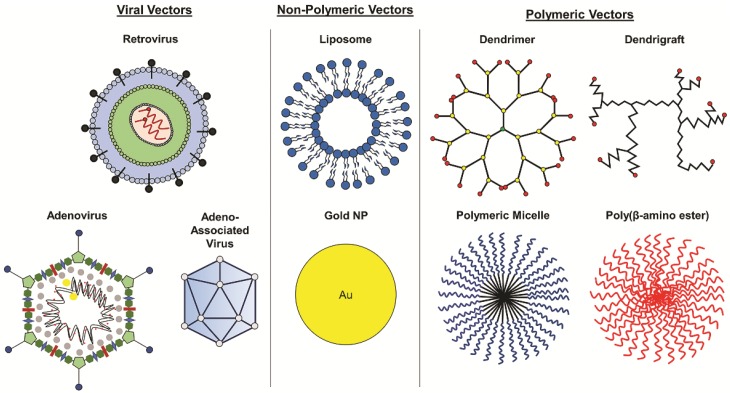
Vectors for glioblastoma gene therapy. Various viral, non-polymeric, and polymeric vectors are used to deliver nucleic acids for GBM gene therapy.

Gene therapy for cancer treatment conventionally includes the introduction of growth regulating or tumor suppressing genes. More recently, RNA interference (RNAi) has been introduced to inhibit the activity of oncogenes causing tumorigenesis or proliferation. Suicide gene therapy is another approach that is commonly used in viral gene therapy to convert non-toxic prodrugs into lethal active compounds. Other approaches include oncolytic and immunomodulatory gene therapy [41]. Gene or nucleic acid carriers must be used to deliver these therapies successfully into tumor tissue and have been extensively studied. Delivery vectors such as viral vectors, non-polymeric NPs, and polymeric NPs that have been used in GBM gene therapy are discussed in detail in the following sections (Figure 3).

### 4.1. Viral Vectors

Viral vectors were the first delivery vehicles used for gene therapy in glioma clinical trials and have been studied for glioma gene therapy over the past 25 years. Viral vectors commonly used for GBM gene therapy in clinical trials include neurotropic retroviruses [42] and adenoviruses [43] that are able to infect neurons and glial cells, such as herpes simplex virus-1 (HSV-1) [44]. Adeno-associated viruses have recently shown promise for gene therapy in treating gliomas in preclinical trials, but have not yet been evaluated in clinical trials [45,46,47]. Current and completed clinical trials using various vectors for gene therapy in glioblastoma treatment have been outlined in Table 2.

Retroviral vectors were the first delivery systems evaluated in clinical trials for glioma gene therapy. The initial trial evaluated the combination of modified murine cells containing retroviral herpes simplex virus-thymidine kinase (HSV-tk) with ganciclovir (Cytovene) and began in 1992 (NCT00001328). HSV-tk functions as a suicide gene and converts the prodrug, ganciclovir, into its active form, ganciclovir-triphosphate, which inhibits DNA replication and cell division in HSV-tk-transfected cells [48]. The results of the study demonstrated intratumorally implanted retroviral vector-producing cells mediated HSV-tk transfection and antitumor activity only in the smaller treated tumors [49], reflecting the limited transfection efficiency of the retroviral vector. Another retroviral vector, Toca 511 delivers suicide gene, cytosine deaminase (CD), and in combination with oral prodrug, Toca FC, the CD enzyme mediates conversion of 5-fluorocytosine into the active antineoplastic drug, 5-fluorouracil [50,51]. Phase I clinical trials demonstrated that Toca 511 and Toca FC were well tolerated and mediated tumor regression in the infusion site in patients with recurrent high-grade glioma [52]. Toca 511 and Toca FC currently make up a regimen in phase 2/3 clinical trials for the treatment of GBM and anaplastic astrocytoma. 

Adenoviral vectors have also been widely evaluated in clinical trials. A phase 1 trial of an adenoviral vector carrying the wild-type p53 gene (Ad-p53) demonstrated that Ad-p53 successfully transfected astrocytic tumor cells with minimal toxicity when intratumorally injected pre- and post-resection of the glioma tumor; however, transfected cells were only detected on average within 5 mm of the injection site [53], demonstrating the limited ability of the therapeutic to penetrate the tumor tissue. Another study compared combination therapy using ganciclovir and intratumoral injection of HSV-tk delivered either by retrovirus-packaging cells or adenoviruses in patients with malignant glioma. The results revealed stable tumor in 3/7 patients treated with the adenovirus compared to tumor progression in all patients treated with the retrovirus three months post-treatment [54]. Additionally, survival time nearly doubled in patients treated with adenovirus compared to retrovirus, with averages of 15 months and 7.4 months, respectively [54]. Sandmair et al. concluded that ineffectiveness of retroviruses may be due to low transfection and brain tumor penetration [54]. Several clinical studies have also evaluated the delivery of an adenoviral vector containing HSV-tk (AdV-tk) combined with valacyclovir, an antiherpetic prodrug. Using gene-mediated cytotoxic immunotherapy, thymidine kinase mediates conversion of the prodrug into toxic nucleotide analogs, inducing tumor cell death and activation of antitumor immune cells [55]. A Phase 1B study of AdV-tk with concurrent valacyclovir and radiation therapy followed by TMZ was conducted in patients with recently diagnosed malignant glioma [55]. AdV-tk injected into the tumor bed post-resection followed by radiation and chemotherapy resulted in two and three-year survival rates of 33% and 25% [55], respectively, a small increase over the current standard of care. Of note, CD3^+^ T-cells were found in tumors analyzed post-treatment [55], indicative of immune activation. A phase 2 trial showed that median survival time significantly increased from 13.5 months for patients that received the standard of care treatment to 17.1 months for patients treated with AdV-tk combined with valacyclovir and standard of care [56]. Additionally, a Phase I trial of AdV-tk with combination valacyclovir and radiation therapy was recently conducted in pediatric malignant glioma and recurrent ependymoma [57]. Half of the patients survived at least 16 months post-treatment with no dose-limited toxicities, though grade 3 lymphopenia was common [57].

Although viral vectors have been studied extensively, they have only resulted in marginal increases in overall survival and have yet to achieve clinical translation through FDA approval to treat patients with GBM after decades of study. Efficient tumor penetration of viral vectors has proven to be a challenge limiting overall efficacy in treating gliomas. However, there has been some clinical success with viral vectors in other cancers. Talimogene laherparepvec is an oncolytic virotherapy consisting of genetically modified HSV-1 containing the human granulocyte-macrophage colony-stimulating factor (GM-CSF) gene and has been FDA approved for the treatment of melanoma [58]. 

### 4.2. Non-Viral Vectors

In addition to viral vectors, non-viral vectors, including both non-polymeric and polymeric delivery systems (Figure 3), have been explored for delivery of glioma gene therapy and have shown promise as gene vectors for glioma treatment in preclinical and clinical studies. Though these vectors have yet to achieve FDA approval for treatment of GBM, the 2018 approval of the first RNAi therapeutic, Patisiran, a lipid nanoparticle containing siRNAs for the treatment of transthyretin-mediated amyloidosis [59], a neurodegenerative disease, provides a promising outlook for non-viral vector-based nucleic acid therapies. A few non-polymeric vectors have been evaluated clinically for GBM gene therapy, including liposomes, gold nanoparticles, and RNA nanoparticles. SGT-53, a transferrin receptor-targeted liposomal vector encapsulating wild-type p53 plasmid DNA is able to cross the BBB and target GBM cells, resulting in a reduction of MGMT and apoptosis in GBM xenografts in mice [60]. Combination therapy with systemically administered SGT-53 and TMZ enhanced antitumor efficacy compared to TMZ alone [60,61], demonstrating the ability of SGT-53 to improve chemosensitivity. SGT-53 is currently in phase II clinical trials for combination therapy with TMZ in treating recurrent glioblastoma (see Table 2). Additionally, NU-0129, a spherical nucleic acid gold nanoparticle containing siRNAs targeting Bcl-2-like protein 12 (Bcl2L12), which is involved in tumor progression and resistance to apoptosis [62], is in early phase 1 clinical trials for patients with recurrent glioblastoma or gliosarcoma. NU-0129 has previously demonstrated its ability to cross the BBB in xenograft models of GBM in mice after systemic administration, resulting in increased apoptosis of tumor cells and reduced tumor progression [62]. In addition to clinical studies, novel polymeric vectors are being explored in research, such as RNA nanoparticles. RNA nanoparticles completely composed of RNA have been used in preclinical studies for glioma gene therapy. Croce et al. reported using RNA nanoparticles to deliver anti-miR-21 locked nucleic acid sequences to inhibit oncogenic miR-21 in xenograft GBM models in mice, resulting in tumor regression and increased survival compared to untreated mice [63]. Though still in their preclinical stages of development, RNA nanoparticles have shown promise for gene delivery in cancer treatment [64].

### 4.3. Polymeric Delivery Systems

Polymeric delivery of gene therapy is an emerging approach for cancer treatment to improve therapeutic outcomes. Current research has been focused on micro- and nanoparticles for the systemic or local delivery of genes and/or drugs. These NPs are advantageous for gene therapy because they are highly tailorable, allowing for conjugation of nucleic acids, homing peptides, or targeting ligands. Though they have not yet reached clinical trials specifically for glioblastoma treatment, several polymeric delivery systems have been studied for use in gene therapy for glioma treatment, and are discussed in detail as follows, including their advantages and limitations. The advantages and disadvantages of various vectors that have been studied for glioblastoma gene therapy are summarized in Table 3.

#### 4.3.1. Dendrimers

Dendrimers are highly branched 3D polymers that have been explored for a variety of applications in drug and gene delivery. Cationic dendrimers, such as poly(amidoamine) (PAMAM), are particularly useful for gene therapy in glioma treatment due to their ability to form complexes with negatively charged nucleic acids, penetrate cellular and endosomal membranes, and cross the BBB. Dendrimers have been used to deliver several types of nucleic acids, including antisense oligonucleotides [65], microRNAs [66], siRNAs [67], and genes [68,69,70,71] into glioma cells. Functionalized dendrimers have demonstrated a capacity for enhanced transfection and targeted delivery in glioma cells and tissues. Specifically, peptide functionalized dendrimers have been used to increase gene transfection in patient-derived primary glioma cells. Bae et al. showed that PAMAM dendrimers grafted with histidine and arginine residues enhanced transfection efficiency in glioma cells compared to PAMAM alone [68]. This result is likely due to the increased proton buffering capacity provided by the peptides, resulting in enhanced endosomal escape and gene transfection. Additionally, several groups have used PAMAM for targeted delivery of gene therapy by functionalizing the polymer with polyethylene glycol (PEG) to attach a targeting moiety. PAMAM-PEG conjugated with transferrin [69], chlorotoxin [70], or Angiopep-2 [71] have allowed increased distribution of therapeutics in glioma tissue after systemic delivery in mice or rats in comparison to treatment with PAMAM-PEG alone, demonstrating the clinical potential of ligand-conjugated dendrimers for intravenous delivery of gene therapy for glioma treatment. However, one of the critical limitations of PAMAM dendrimers for clinical translation is cytotoxicity due to their high positive surface charge. Studies have shown that PAMAM dendrimers exhibit neurotoxicity by inducing autophagy in glioma cells, resulting in cell death [72]. Strategies to mitigate this effect include reducing the surface charge through acetylation or functionalization using PEG.

#### 4.3.2. Dendrigraft

Similar to dendrimers, dendrigrafts are also dendritic structures that can be used to deliver therapeutics. Dendrigraft poly-l-lysine (DGL) was recently discovered as a newer class of dendritic polymers and has shown potential for delivery of nucleic acids. One major advantage of DGL over dendrimers is that DGL is composed entirely of naturally occurring lysine residues and is therefore completely biodegradable. Dendrigrafts are rich in external amino groups, which enables self-assembly with nucleic acids. DGL is also non-immunogenic and has been demonstrated to cross the BBB [73]. To mediate glioma targeting, transferrin- [74] or laminin-targeted [75] peptides have been conjugated to DGL for gene therapy with pORF-hTRAIL or survivin, respectively. The results of both studies revealed that DGL conjugated with targeting peptides exhibits enhanced tumor targeting and long-term survival in xenograft mouse models of U87 human glioblastoma in comparison to non-targeted DGL [74,75]. Transferrin-targeted DGL has also been used successfully for RNAi therapy. Kuang et al. demonstrated that transferrin-targeted DGL mediates increased gene silencing in mouse glioma tissue in comparison to non-targeted DGL [76]. In another study, a cell-penetrating peptide conjugated to DGL for delivery of pcDNA3.1-ING4, a plasmid encoding tumor suppressor gene inhibitor of growth 4 (ING4), demonstrated enhanced apoptosis of U87 tumor cells and resulted in increased survival of mice in comparison to treatment with DGL/pDNA [77]. DGL has also been used for combination delivery of a drug and gene. Li et al. demonstrated that choline-targeted DGL delivers pORF-hTRAIL and doxorubicin to glioma tissue in mice [78]. Similar to dendrimers, the cytotoxicity of DGL is a major disadvantage due to its excessive cationic charge. Studies have shown that the cytotoxicity of DGL/nucleic acid complexes increases in a dose-dependent manner and also results in hemotoxicity [79,80]. To overcome this limitation, toxicity of DGL can be reduced by including anionic polymers [80] or through PEGylation [81]. Though studies with dendrigrafts are relatively new and still evolving, data thus far has shown their potential clinical applicability for nucleic acid delivery in the treatment of gliomas.

#### 4.3.3. Polymeric Micelles

Polymeric micelles are amphiphilic copolymers that have a core/shell structure. They have been widely used for cancer drug delivery [82], but have recently been explored for delivery of nucleic acids and shown promise for treatment of gliomas. Cationic polymers, such as polyethyleneimine (PEI), are commonly combined with hydrophobic polymers for combination delivery of negatively charged nucleic acids and hydrophobic cancer drugs. Cheng et al. demonstrated that a folate (FA)-targeted micelle consisting of PEI and polycaprolactone (PCL) mediated co-delivery of BCL-2 siRNA and doxorubicin in C6 glioma tumors in rats, resulting in increased apoptosis and inhibition of tumor growth following intratumoral injection [83]. In another study, to reduce the cytotoxic effect of PEI as well as enable active targeting, FA- was conjugated to hyperbranched PEI (FA-PEG-PEI) using PEG as spacer for combination gene therapy with CD/5-FC and TRAIL [84]. The results showed increased anticancer activity in C6 glioma tissue in rats after intratumoral delivery compared to treatment with a single therapeutic [84]. An RGD-conjugated PEI-PEG micelle used to co-deliver pORF-hTRAIL and paclitaxel in mice with orthotopic glioblastoma significantly enhanced survival in comparison to mice treated with gene therapy alone [85]. Additionally, intravenous delivery of PEI-PEG conjugated to a tumor homing peptide targeting neuropilin-1, retro-inverso C-end rule (CendR) peptide D(RPPREGR), enhanced gene transfection efficiency in mice with U87 glioma over non-targeted PEG-PEI [86]. Although PEGylation of PEI reduces cytotoxic effects, it can also reduce transfection efficiency by hindering proton buffering capacity. To overcome this limitation, PEI can be reversibly shielded using degradable disulfide (SS) linkages. Lei et al. conjugated RGD peptide to PEI through PEG using a reversible disulfide linkage (RGD-PEG-SS-PEI) for treatment of mice with U87 glioblastoma [87]. Results showed that the reversibly shielded PEI increased gene expression in the mouse brain in comparison to irreversibly shielded RGD-PEG-PEI [87], demonstrating the potential of reversible shielding for reducing cytotoxicity of PEI while maintaining efficient transfection.

#### 4.3.4. Poly(β-amino ester)

Poly(β-amino esters) (PBAEs) are another class of cationic polymer that were designed to meet specific criteria for gene delivery, including DNA condensation, biodegradability, and minimal cytotoxicity [88], PBAEs may contain different types of amines and can be synthesized to create large libraries of polymers using combinatorial chemistry, allowing high-throughput screening of hundreds of polymers and identification of optimal vectors for gene delivery [89]. Research has shown that optimal PBAE vectors can transfect up to 90% of primary GBM cells and mediate up to 85% gene silencing with minimal cytotoxicity [90]. Further, PBAEs have been used as vectors for local injection of therapeutics. PBAE nanoparticles have been proven to penetrate glioma tissue for gene delivery using various strategies. For example, intratumoral injection was used to deliver DNA-containing PBAE nanoparticles in rat models of 9L gliosarcoma [91]. Convection-enhanced delivery (CED) is another local delivery strategy that allows administration of a therapeutic into glioma tumor tissue through catheters placed directly in the tissue with infusion occurring over the course of several hours. CED has been used for PBAE-mediated DNA delivery combined with intraperitoneal administration of ganciclovir in mouse xenograft models of primary brain tumor-initiating cells [92]. To enhance brain penetration, modification of PBAE nanoparticles with PEG has been explored. Mastorakos et al. demonstrated that PEGylated PBAEs could penetrate brain tissue with 20-fold greater volume distribution following CED in comparison to non-PEGylated particles [93]. One of the disadvantages of PBAEs involves their mechanism for cargo release. PBAEs release their cargo through hydrolysis of ester bonds, which can occur over many hours to a couple days [94], resulting in lack of controlled release of therapeutics. To overcome this limitation, bioreducible PBAEs have been synthesized containing disulfide bonds with the ability to trigger release of siRNAs into the cytoplasm [95]. Thus far, PBAEs have shown promise in overcoming limited tissue distribution, a common barrier in clinical applications of local gene therapy. Further research demonstrating enhanced tissue-penetration using PEGylated PBAEs in a glioma model will allow further assessment of the clinical potential of PEGylated PBAEs for local gene therapy of gliomas.

## 5. Conclusions

Glioblastoma multiforme is a common and currently incurable brain cancer that desperately needs new treatment modalities to improve patient outcomes. The current standard of care including surgical resection, adjuvant chemotherapy, and radiation does not result in remission for the majority of patients. Barriers limiting efficacy include inefficient delivery across the BBB and therapeutic resistance. Gene therapy represents an approach to specifically target and regulate oncogenes and tumor suppressor gene in gliomas. Further, gene therapy can be used to overcome barriers such as chemotherapy resistance by downregulating resistance genes or using approaches such as suicide gene therapy. Viral vectors, including retroviruses and adenoviruses, have been evaluated in clinical trials of GBM for the past few decades for delivery of therapeutic genes or nucleic acids in combination with other therapeutics. However, viral vectors have not reached clinical approval due to immunogenicity, limited tumor penetration and marginal improvement in patient outcomes. Non-viral delivery is an evolving alternative approach that may be used to overcome the barriers of gene delivery. Many non-viral vectors, including polymeric and non-polymeric vectors, are non-immunogenic and can be functionalized with targeting moieties to increase receptor-mediated uptake of vectors into tumor tissue. Multifunctional and multimodal non-polymeric vectors, such as liposomes and gold NPs, respectively, have the ability to co-deliver multiple therapies or be used for tumor imaging as well as therapy. Cationic polymeric vectors have the ability to self-assemble with nucleic acids, enhancing their ease of use for gene therapy over other vectors. Moreover, polymeric vectors such as PBAE, have demonstrated potential for improving tissue penetration, one of the largest barriers to increasing efficacy of gene therapy vectors in glioblastoma. To date, only a few non-viral vectors have been evaluated in clinical trials for GBM; however, further evaluation of non-viral vectors in clinical trials in the future may provide advanced treatment strategies for gene therapy in glioblastoma.

## Figures and Tables

**Table 1:** Gene expression in glioblastoma multiforme (GBM). Common gene targets that are mutated or upregulated in glioblastoma.

Gene Target	Effect	GBM Clinical Subtype	References
EGFR (epidermal growth factor receptor)	Reduction in apoptosis and increased uncontrolled cell proliferation	Classical	[13,14]
PTEN (phosphate and tensin homologue)	Activation of the P13K/Akt/mTOR pathway, leading to cell proliferation, migration and growth	Classical	[1,13,15,16]
PDGFRA (platelet derived growth factor receptor—alpha)	Increased tumor cell proliferation	Proneural	[11,13,14]
IDH-1 (isocitrate dehydrogenase 1)	Alters DNA and histone methylation	Proneural	[17,18]
Tumor suppressor p53	Uncontrolled cell growth	Proneural, mesenchymal	[13,14,20]
NF-1 (neurofibromin 1)	Uncontrolled cell growth	Mesenchymal	[13]

**Table 2:** Vectors for glioma gene therapy. Vectors that have been evaluated in clinical trials for glioma gene therapy.

Vector	Gene Therapy Agent	Mechanism	Combination Therapy	Clinical Trial Phase	Clinical Trial Number
**Retrovirus**	HSV-tk	Suicide gene therapy, HSV-tk converts ganciclovir to antiviral drug ganciclovir triphosphate	Ganciclovir	Phase I	NCT00001328
**Retrovirus**	Toca 511	Suicide gene therapy, CD converts prodrug 5-FC to anti-neoplastic 5-FU	Oral 5-FC	Phase II/III	NCT02414165
**Adenovirus**	SCH-58500	Tumor suppressor gene therapy, transfects p53 gene	N/A	Phase I	NCT00004080
**Adenovirus**	Ad-p53	Tumor suppressor gene therapy, transfects p53 gene	N/A	Phase I	NCT00004041
**Retro or adenovirus**	HSV-tk	Suicide gene therapy, HSV-tk converts ganciclovir to antiviral drug ganciclovir triphosphate	Ganciclovir	Phase I	Sandmair et. al.
**Adenovirus**	AdV-tk	Gene-mediated cytotoxic immunotherapy, HSV-tk converts valacyclovir to antiviral drug acyclovir	Valacyclovir	Phase I	NCT00751270
**Adenovirus**	AdV-tk	Gene-mediated cytotoxic immunotherapy, HSV-tk converts valacyclovir to antiviral drug acyclovir	Valacyclovir and radiation therapy	Phase IIa	NCT00589875
**Liposome**	SGT-53	Tumor suppressor gene therapy, transfects p53 gene	TMZ	Phase II	NCT02340156
**Spherical Nucleic Acid Gold NP**	NU-0129	RNAi gene therapy, transfects siRNAs targeting oncogene Bcl2L12	N/A	Early Phase I	NCT03020017

**Table 3:** Comparison of gene delivery vectors. Advantages and disadvantages of various vectors for glioblastoma gene therapy.

Vector	Advantages	Disadvantages
Viral		
Adenovirus	Deliver large DNA	Transient gene expression Elicit immune response
Retrovirus	Transfer to dividing cells Sustained expression of vector	Elicit immune response Unable to transfect non-dividing cells Low transfection rate in vivo Risk of insertion
Adeno-associated virus	Transfer to dividing and non-dividing cells	Difficult to produce vectors Limited transgene capacity Elicit immune response
Non-Viral		
Liposome	Non-immunogenic Ability to co-deliver gene therapy and chemotherapy Ability to functionalize for targeting	Short shelf- and half-life Transient gene expression Low transfection efficiency Increased cytotoxicity for cationic lipids
Gold nanoparticles	Multimodal use for tumor imaging and therapy Ability to functionalize for targeting	Non-biodegradable
Dendrimer & Dendrigraft	Self-assemble with nucleic acids Ability to functionalize for targeting Non-immunogenic	Increased cytotoxicity for cationic dendrimers Limited release of therapeutics
Polymeric micelles	Self-assemble with nucleic acids Ability to functionalize for targeting	Increased cytotoxicity for PEI and other cationic polymers Low loading efficiency
Poly(β-amino ester)	Biodegradable Lower cytotoxicity than other cationic polymers High transfection efficiency	Limited control over release of therapeutics
